# Data on eye movements in people with glaucoma and peers with normal vision

**DOI:** 10.1016/j.dib.2018.05.076

**Published:** 2018-05-18

**Authors:** Daniel S. Asfaw, Pete R. Jones, Nicholas D. Smith, David P. Crabb

**Affiliations:** Division of Optometry and Visual Science, School of Health Science, City, University of London, London, EC1V 0HB, UK

**Keywords:** Eye movements, Scanpaths, Visual fields, Glaucoma, Eye tracking

## Abstract

Eye movements of glaucoma patients have been shown to differ from age-similar control groups when performing everyday tasks, such as reading (Burton et al., 2012; Smith et al., 2014) [1], [2], visual search (Smith et al., 2012) [3], face recognition (Glen et al., 2013) [4], driving, and viewing static images (Smith et al., 2012) [5]. Described here is the dataset from a recent publication in which we compared the eye-movements of 44 glaucoma patients and 32 age-similar controls, while they watched a series of short video clips taken from television programs (Crabb et al., 2018) [6]. Gaze was recorded at 1000 Hz using a remote eye-tracker. We also provide demographic information and results from a clinical examination of vision for each participant.

**Specifications Table**

**Table:** 

Subject area	Visual science
More specific subject area	Visual science, Optometry, Statistics
Type of data	Table (csv file) and raw data (ASCII text format)
How data was acquired	Monocular eye movements were recorded using the EyeLink 1000 (SR Research Ltd., Ontario, Canada) eye tracker. Visual field data were acquired using Humphrey Field Analyzer (HFA; Carl Zeiss Meditec, CA, USA). Visual acuity was measured using an Early Treatment Diabetic Retinopathy Study (ETDRS) chart and contrast sensitivity was measured with a Pelli-Robson chart.
Data format	Raw data, analyzed
Experimental factors	Participant (44 glaucoma patients and 32 peers with normal vision) watched three separate video clips without any explicit task instruction.
Experimental features	Participants were positioned, using a chin rest, at a viewing distance of 60 cm.
Data source location	School of Health Science, City, University of London, UK
Data accessibility	The dataset is freely available (at https://doi.org/10.5281/zenodo.1156863) for any academic, educational, and research purposes.

**Value of the data**

• Raw eye tracking data from 76 people with a median (interquartile range) age of 68 (63, 75) years will be useful for reanalysis by other scholars.
• The data will allow researchers to develop their own methods for assessing eye movements while people watch everyday videos.
• Data from clinical examinations of vision (visual acuity, contrast sensitivity, and visual field loss) could be used to investigate the relationship between eye movements and vision loss.
• Data from visual fields could be used to explore the relationship between glaucoma and eye movements.

## 1 Data

Eye movement data were collected to test the hypothesis that age-related neurodegenerative eye disease can be detected in a person׳s spontaneous eye-movements while watching video clips [6]. Gaze was recorded in 44 glaucoma patients, and 32 age-similar people with healthy vision. All patients had an established clinical diagnosis of chronic open angle glaucoma (COAG): an age-related disease of the optic nerve that can result in a progressive loss of visual function [7], [8], Each participant watched three video clips, for approximately 16 min in total, and completed standard clinical tests of visual function (visual acuity, contrast sensitivity, visual field examination). The dataset contains raw gaze data, processed eye movement data, clinical vision test results, and basic demographic information (age, sex) [1], [2], [3], [4], [5].

### 1.1 Participants

Forty-four people with glaucoma were recruited from clinics at Moorfields Eye Hospital NHS Foundation Trust, London. All patients had an established clinical diagnosis of chronic open angle glaucoma (COAG) for at least two years and were between 50 and 80 years of age. COAG was defined, following clinical guidelines, by the presence of reproducible visual field defects in at least one eye with corresponding damage to the optic nerve head and an open iridocorneal drainage angle on gonioscopy [9]. The diagnosis was made by a glaucoma specialist. A deliberate attempt was made to recruit a sample of patients with a range of disease severity according to visual field loss. Patients were purposely not recruited if they had any ocular disease other than glaucoma (except for an uncomplicated lens replacement cataract surgery). In addition, at the point of recruitment, patients had slit lamp biomicroscopy performed by an ophthalmologist to further exclude any other concomitant macular pathology, ocular surface disease or any significant problems with dry eye.

Thirty two healthy people (controls), of a similar age to the patients, were recruited from the City University London Optometry Clinic; this is a primary care centre where people routinely receive a full eye examination, which includes measurement of visual acuity, refraction, binocular vision assessment, pupil reactions, slit-lamp assessment of the anterior eye, measurement of intraocular pressure, visual field assessment and indirect ophthalmoscopy of the macula, optic nerve head, and peripheral retina.

### 1.2 Clinical vision tests

All participants underwent an examination of visual function by a qualified optometrist on the day of testing. Corrected binocular visual acuity (VA) was measured using an Early Treatment Diabetic Retinopathy Study (ETDRS) letter chart. All participants had binocular VA of at least 0.18 logMAR (Snellen equivalent of 6/9). Binocular Contrast Sensitivity (CS) was measured with a Pelli-Robson chart. Visual fields were measured monocularly in both eyes using automated static threshold perimetry. This was performed using a Humphrey Field Analyzer (HFA; Carl Zeiss Meditec, CA, USA), with a standard 24-2 grid and the Swedish Interactive Testing Algorithm (SITA). HFA mean deviation (MD) is a standard measure of overall visual field loss, relative to healthy age-matched observers, with more negative values indicating greater loss. The Oculus C-Quant straylight meter (Oculus GmbH, Wetzlar, Germany) was used to measure abnormal light scattering in the eye media, to exclude participants with media opacity and other lens type artifacts. Participants were required to be within “normal limits” for this test. Furthermore, all participants were examined with a modified version of the Middlesex Elderly Assessment of Mental State (MEAMS, Pearson, London, UK), a psychometric test designed to detect gross impairment of specific cognitive skills such as memory and object recognition in an elderly population. All participants passed the MEAMS test. The light scattering and MEAMS tests results are not included in the hosted data; however, VA, CS, and visual field data are included.

Summary measures of these vision tests, such as HFA MD in decibels (dB), visual acuity (VA) in logMAR, and contrast sensitivity (CS) in log units are provided in a single comma-separated file, along with basic demographic information. A sample of these data is shown in Table 1. These data allow investigating the relationship between different eye movement parameters (such as saccade amplitudes and rates) and common clinical measures of vision.

**Table 1:** Sample clinical information of participants. The complete tables for both patients and controls are uploaded in a spreadsheet file. The tables have eight fields: participants’ ID, the eye used for the study, age, sex, MD measurements (for both left and right eyes), binocular VA, and CS measurements. Participants were assigned a unique ID, G001 – G044 for patients and C001 – C032 for controls. Shown here are the data from the first five patients.

**Participant ID**	**Study eye**	**Age**	**Sex**	**Left MD**	**Right MD**	**VA (log)**	**Log CS**
**G001**	L	**63**	**Female**	−20.84	−6.1	-0.02	1.95
**G002**	L	**69**	**Female**	−8.17	−12.05	0.04	1.95
**G003**	L	**77**	**Female**	−3.61	−2.24	0.16	1.95
**G004**	L	**74**	**Male**	−10.42	−4.66	0.14	1.95
**G005**	L	**64**	**Male**	−3.56	−6.45	0.02	1.65

**Fig. 1:**
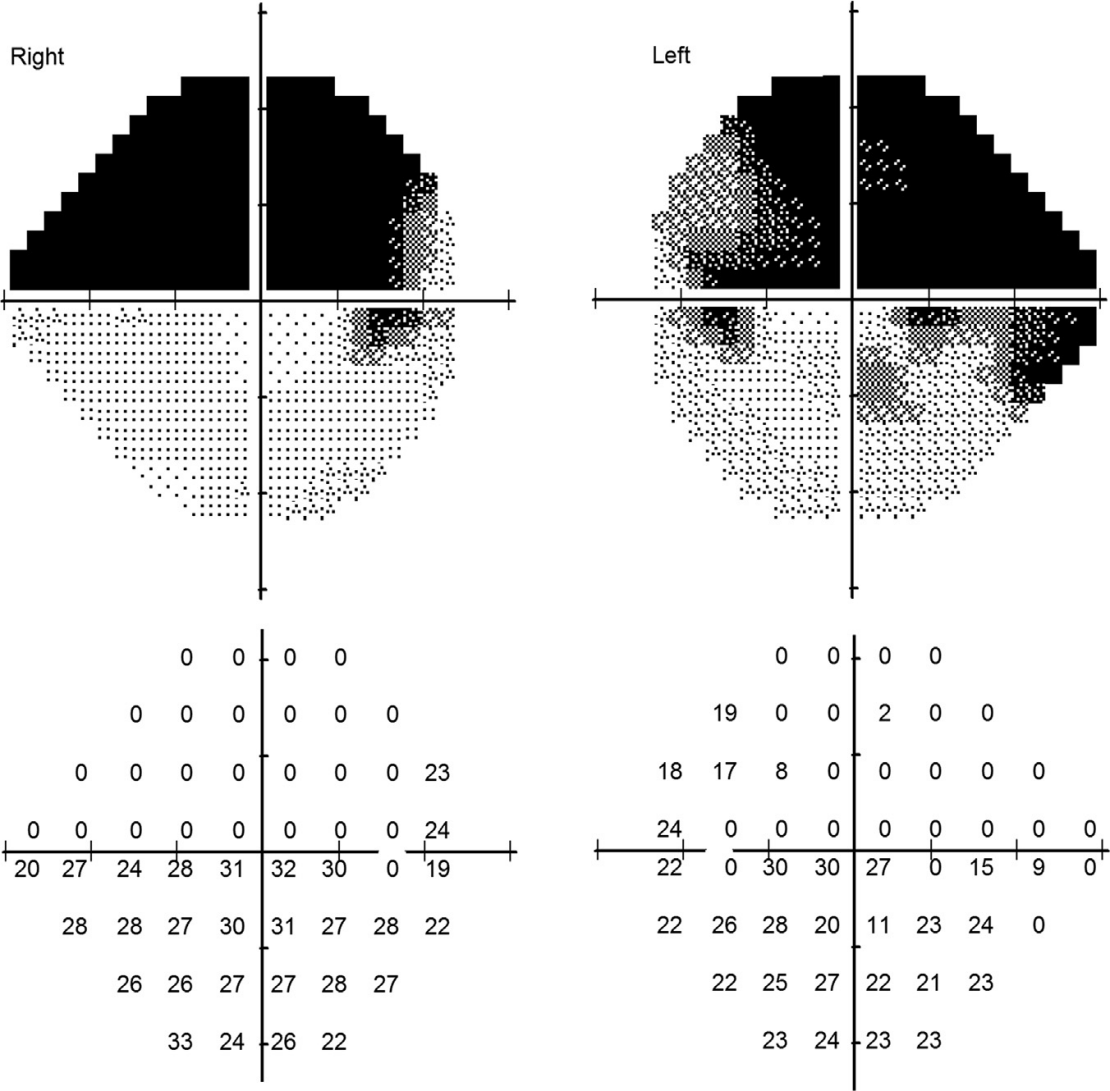
Sample 24-2 visual field grey scale plots and the corresponding numeric visual field map (for participant G007). The 54 sensitivity values in the visual field map are vectorized and stored in comma separated file (see Table 2). The vectorization was performed by concatenating sensitivity values starting from first row (top) to the last row (bottom). The same vectorization procedure was applied to sensitivity values of both eyes.

Individual Differential Light Sensitivity (DLS) values [10] for each of the 54 test points in the 24-2 visual field test are provided for every participant/eye. These values are stored in a single row, as shown in Table 2 and can be visualized in visual field map as shown in Fig. 1. These data could be used to investigate the effect of field loss on eye movements; for example, in the past there have been attempts to relate the directions of spontaneous saccades to locations of visual field loss [11], [12].

**Table 2:** Sample sensitivity values for each of the 54 test points in the 24-2 visual field test. The results provided are for every participant/eye. The data for G007 is also shown graphically in Fig. 1.

***Participant***	***Eye***										
**G001**	LEFT	27	22	28	23	29	*…*	29	29	28	21
**G001**	RIGHT	11	10	7	0	6	*…*	10	23	25	23
**…**	….										
**G007**	LEFT	0	0	0	0	19	*…*	23	24	23	23
**G007**	RIGHT	0	0	0	0	0	*…*	33	24	26	22
**….**	….										
**G044**	LEFT	26	26	25	19	25	…	31	31	28	27
**G044**	RIGHT	28	28	19	23	28	*…*	27	28	28	26

### 1.3 Raw gaze data

Gaze was measured using an Eyelink 1000 eye-tracker (SR Research Ltd., Ontario, Canada). Participants were positioned, using a chin rest, at a viewing distance of 60 cm. The eye-tracker outputs data in a proprietary EDF file format (.EDF; Eyelink Data File). For ease of use, these EDF files were converted into ASC file using a translator program (EDF2ASC) that was supplied by SR-Research. The ASC files contain the entire recorded eye tracking events, including the start and end time of all the eye movement events such as fixations, saccades, and blinks. During fixations and saccades, the eye position (in screen coordinate) was recorded. Other eye tracking information such as calibration and synchronization information were also stored in the ASC files. The gaze data for each participant were stored in individual ASC files (i.e., 44 and 32 ASC files for glaucoma and controls, respectively). Detailed description of the ASC file׳s format and structure are provided by the manufacturer (SR Research; https://www.sr-research.com).

### 1.4 Processed eye movement data

We processed the raw ASC file to extract fixations and saccades using a bespoke C++ program. The program searches for flags that indicate the end of a fixation (‘EFIX’) or a saccade (‘ESACC’) in the ASC file. Each fixation end flag contains its start and end time, duration, mean position, and mean pupil size during the fixation. Similarly, a saccade end flag contains its amplitude, velocity, duration, start and end time, and start and end position. The extracted fixation and saccade events have eight and eleven fields, respectively (Table 3). These processed eye movement data were stored in CSV file format. Thus, the dataset contains 44 and 32 CSV files for glaucoma and controls, respectively. It should be noted that due to poor tracking and technical errors, the data from five controls (C019 – C023) and one patient (G010) are incomplete. Their data, however, are included in the dataset for completeness.

**Table 3:** Description of fixation and saccade fields contained within the “processed eye-movement data” CSV files. Five events (trial name, eye, start time, end time, and duration) are similar for both fixation and saccade events. Saccade and fixation positions are expressed using four (Start *X*, Start *Y*, End *X*, and End *Y*) and two (*X* and *Y*) fields, respectively. In addition, each saccade has two additional fields that describe the size and speed of the saccade.

Field	Description
Trial name	Name of video (one of ‘DadsArmy’,’HistoryBoys’, and ‘SkiCross’; see Fig. 2)
Eye	The study eye (left or right)
Start time	Start time of the event (e.g., saccade start, saccade end; in millisecond)
End time	End time of the event (in millisecond)
Duration	Difference between the start and end of the event in millisecond
*X*	The *x* position of fixation in screen coordinate in pixels (range from 1 to 1600)
*Y*	The *y* position of fixation in screen coordinate in pixels (range from 1 to 1200)
Pupil area	Pupil area of the eye during fixation
Start X	The x coordinate of saccade׳s starting position in pixels (range from 1 to 1600)
Start Y	The y coordinate of saccade׳s starting position in pixels (range from 1 to 1200)
End *X*	The *x* coordinate of saccade׳s end position in pixels (range from 1 to 1600)
End *Y*	The *y* coordinate of saccade׳s end position in pixels (range from 1 to 1200)
Amplitude	Size of the saccade in degrees visual angle
Peak Velocity	Speed of the saccade in degrees/second

Within the data archive, we include a Minimal Working Example MATLAB script (‘SaccadeAmplitudePlot.m’) which demonstrates how this processed data can be used (in this case, to plot the distribution of saccade amplitude of each participant). This program can be extended easily to compute other eye movement metrics, such as fixation duration or saccade rate, that are commonly used to quantify the visual behaviors of patients and controls [1], [2], [3], [4], [5].

## 2 Experimental design, materials and methods

### 2.1 Apparatus

Participants viewed sequentially three unmodified TV and film clips (including audio) on a 22 in. monitor (Iiyama Vision Master PRO 514, Iiyama Corporation, Tokyo, Japan) at a resolution of 1600 by 1200 pixels (refresh rate 100 Hz). Monocular eye movements were recorded using an Eyelink 1000 eye tracker (SR Research, Ontario, Canada), while participants watched the video clips monocularly. The eye tracker was configured to detect saccades using velocity and acceleration thresholds of 30°/s and 8000°/s^2^, respectively. The eye giving the best quality pupil detection and corneal reflection was chosen for tracking. The EyeLink proprietary algorithm (nine point calibration) was used to calibrate the eye tracker and was repeated, as required, until the accuracy was judged by the software to be of a “good” quality. Drift correction was also performed prior to each of the three video clips. In cases where a large drift (> 5°) was detected, a recalibration was performed.

### 2.2 Stimuli

**Fig. 2:**
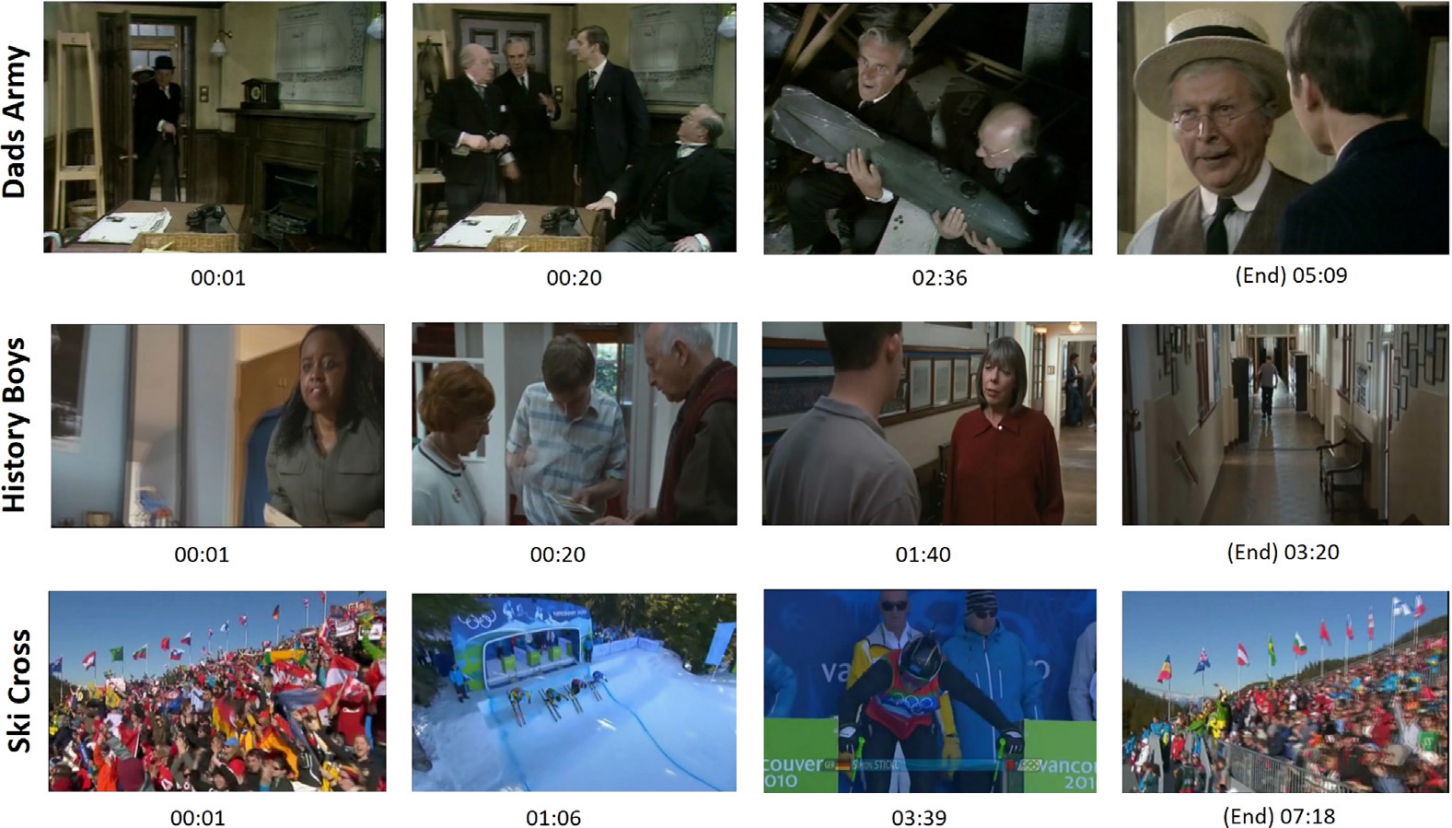
Sample frames excerpted at a specific time from the three video clip used in the experiment.

One clip (top row in Fig. 2) was an excerpt from an entertainment program (309 s; Dads Army, BBC Television) which covered the full screen (subtending a half-angle of 20.3° by 14.9°). The other two clips (middle and bottom rows in Fig. 2) were taken from a feature film (200 s; The History Boys, 20th Century Fox) and a sport program (436 s; 2010 Vancouver Winter Olympics Men׳s Ski Cross, BBC Television); both of these clips were recorded at a 16:9 ratio, therefore they contained black rectangles at the top and bottom of the screen (subtended a half-angle of 17.3° by 10.6°). We summarized the characteristics of the three stimuli in Table 4.

**Table 4:** Details of the stimuli (three video clips) used in the experiment.

**Clip name**	**Subtending angle (a half-angle)**	**Length (minutes: seconds)**	**Frame width (pixels)**	**Frame height (pixels)**	**Frame rate (frames/second)**
**Dads Army**	17.3° × 10.6°	05:09	1280	720	29
**History Boys**	17.3° × 10.6°	03:20	720	576	25
**Ski cross**	17.3° × 10.6°	07:18	720	576	25

### 2.3 Ethics

The study was approved by the Moorfields and Whittington Research Ethics Committee, London and the School of Health Sciences Research and Ethics Committee, City, University of London. Written informed consent, was obtained prior to examination from each participant, and the research was conducted in accordance with the to the tenets of the Declaration of Helsinki.
